# Emerging Biomarkers and Electrochemical Biosensors for Early Detection of Premature Coronary Artery Disease

**DOI:** 10.3390/diagnostics15070940

**Published:** 2025-04-07

**Authors:** Nanthini Mageswaran, Sarah Husnaini Zainal, Nurul Izzaty Hassan, Nurul Huda Abd Karim, Noor Akmal Shareela Ismail

**Affiliations:** 1 Department of Medicine, Faculty of Medicine, Universiti Kebangsaan Malaysia, Kuala Lumpur 56000, Malaysia; mageswaran.nanthini@ukm.edu.my; 2 Department of Biochemistry, Faculty of Medicine, Universiti Kebangsaan Malaysia, Kuala Lumpur 56000, Malaysia; sarahusnaini@gmail.com; 3 Department of Chemical Sciences, Faculty of Science & Technology, Universiti Kebangsaan Malaysia, Bangi 43600, Malaysia; drizz@ukm.edu.my (N.I.H.); nurulhuda@ukm.edu.my (N.H.A.K.)

**Keywords:** biosensor, premature coronary artery disease, electrochemistry, point-of-care testing, serum

## Abstract

Coronary artery disease (CAD) is one of the primary causes of morbidity and death worldwide. Premature CAD (pCAD) is the term used to describe the 3–10% of CAD occurrences that occur in people under 45 worldwide. Diagnostic difficulties arise from the different risk factor profiles of pCAD and late-onset CAD. Better cardiovascular risk prediction in younger populations has been made possible by the development of biomarker detection tools. This can be applied to a diagnostic tool, including electrochemical biosensors, which have been predicted to be instrumental because of their adaptability for point-of-care applications for quicker diagnoses. These biosensors provide efficient, scalable, and reasonably priced solutions for the quick identification and tracking of CAD. Multiplex biomarker detection has been adopted as a viable approach for early diagnosis and risk assessment due to the constraints of using a single biomarker for pCAD diagnosis. Thus, this study looks at current developments in biosensing technology and discusses established and new cardiac biomarker panels for pCAD identification.

## 1. Introduction

Coronary artery disease (CAD) continues to be one of the leading causes of global morbidity and mortality, with low- and middle-income countries shouldering a significant portion of the burden [1]. Even though CAD is primarily linked to elderly persons, 3–10% of incidents globally involve those under 45 [2,3]. The onset of a cardiovascular event before the ages of 45 for men and 55 for women is commonly used to characterize this subgroup of individuals, which is known as premature CAD (pCAD) [4]. According to data from 2018 to 2019, 24.5% of the 20,605 acute coronary syndrome (ACS) patients who were admitted to 24 hospitals in Malaysia were younger than 50. Furthermore, compared to patients listed in the GRACE registry and Euro Heart Survey, Malaysians with ACS typically present at a younger age and have a greater prevalence of cardiovascular risk factors [5,6].

Premature CAD is a dynamic and progressive illness and is frequently associated with suboptimal clinical outcomes. A recent local study showed that there is a significantly high prevalence of newly diagnosed cardiovascular risk factors among young Malaysians, especially in those with a family history of young CAD [7]. It is essential to identify subclinical pCAD early so we can anticipate the risk and subsequently treat it. Nowadays, the current diagnosis of pCAD is made more difficult due to an age-related pattern of risk factors that further affects the severity and features of CAD. This pattern is different from late-onset CAD, which has several risk factor profiles [8,9]. The risk factors of CAD are often linked to hypertension, obesity, a sedentary lifestyle, and diabetes mellitus. Common risk factors for pCAD include smoking, opium use, dyslipidemia, and a familial history of CAD [8,9]. Furthermore, new research indicates that atherosclerotic plaques in people 65 and older are typically longer, with a higher plaque burden, necrotic core, and thicker calcium than in the younger population [10]. The gold standard for anatomical evaluation in CAD diagnosis is invasive coronary angiography, which uses radiation and contrast medium to provide a comprehensive image of the coronary architecture, which is not precise in predicting pCAD. Therefore, continuous technological developments have made biomarkers essential instruments for the early detection and evaluation of pCAD.

Endothelial dysfunction, lipid buildup, inflammation, oxidative stress, and epigenetic changes are all directly linked to the etiology and development of atherosclerosis and CAD [11]. Systemic biomarkers for early CAD prediction include inflammatory mediators released by vascular and inflammatory cells in atherosclerotic plaques, as well as from organs, such as the liver and adipose tissue [12]. For CAD identification, a number of well-established biomarkers have been used extensively, such as brain-type natriuretic peptide (BNP), cardiac troponin-I (cTnI), cardiac troponin-T (cTnT), myoglobin (Myo), creatine phosphokinase MB (CK-MB), and N-terminal proBNP (BNPT). Other indicators, including heart fatty acid-binding protein (H-FABP), myeloperoxidase (MPO), and C-reactive protein (CRP), are frequently used as diagnostic instruments. Furthermore, less specific biomarkers, such as copeptin, glycogen phosphorylase isoenzyme BB (GPBB), exosomal microRNAs (exomiRs), lipoprotein-associated phospholipase A2 (Lp-PLA2), interleukins (IL), TNF-alpha, and D-dimer (DDm), are used to distinguish between subcategories of cardiovascular disease [13,14]. Cardiac biomarkers have been identified using a variety of detection techniques, such as colorimetry, fluorescence, electrochemical, electrical, photoelectrochemical (PEC), chemiluminescence, electrochemiluminescence, and surface-enhanced Raman spectroscopy. Despite their high sensitivity, these techniques are frequently expensive and time-consuming. Since several biomarkers are associated with different disorders, using a single biomarker to diagnose CAD has limits due to its lower specificity. Multiplex biomarker identification has thereby become a more precise and disease-specific method, providing increased dependability for pCAD risk classification and early diagnosis. According to recent research, patients with pCAD exhibit higher levels of sVCAM-1, IL-6, and CRP than patients with late-onset CAD, which signify different biomarker profiles between pCAD and late-onset CAD [15]. Therefore, it is essential to establish a different set of biomarkers for pCAD early detection methods to forecast future cardiovascular events in young, high-risk individuals.

The development of instruments for the quick and accurate identification and measurement of cardiovascular disease (CVD) biomarkers has advanced significantly in recent years. These devices have historically been huge tools kept in centralized laboratories, which has limited their accessibility, especially for low-income and rural people. These vulnerable groups are disproportionately affected by CVDs, which highlights the need for affordable, portable, and user-friendly devices that can measure several biomarkers at the point of care. By removing the obstacles to prompt diagnosis and treatment in marginalized communities, such developments can significantly improve patient outcomes. Interest in assessing biomarker performance and investigating multiplexed panels that can be included in robust and reasonably priced biosensor platforms for point-of-care applications has increased due to the rising prevalence of CVD worldwide and the abundance of biomarkers.

However, no thorough evaluation has specifically addressed biosensing techniques for the detection of premature coronary artery disease (pCAD), as the previous literature only focused on biosensors for cardiac biomarkers with a single-marker analysis [16,17,18,19,20,21]. Hence, the goal of this review is to compile all available information on cardiac biomarker panels, both known and new, for the detection of pCAD. This manuscript will focus on potential methods for the diagnosis and treatment of pCAD through several electrochemical biosensing techniques with a linear range and limit of detection (LOD).

## 2. Biomarkers for Premature Coronary Artery Disease

### 2.1. Established Biomarkers for Premature Coronary Artery Disease

#### 2.1.1. High-Sensitivity Cardiac Troponins (hs-cTn)

Cardiac troponins are the biochemical gold standard for identifying heart damage, according to new data. There are three isoforms of these structural proteins, which are components of the actin-myosin complex: C (cTnC), I (cTnI), and T (cTnT), with cTnI and cTnT being cardiac-specific [22]. Both reversible and irreversible myocardial damage cause the release of troponins into the bloodstream. Membranous bleb development, enhanced cellular wall permeability, proteolytic degradation, and normal myocyte turnover are examples of reversible damage processes. On the contrary, tissue necrosis brought on by apoptosis or hypoxia is the main cause of irreparable damage [23]. The usefulness of cardiac troponins is linked to high-sensitivity (hs-) immunoassays, which are useful for cardiovascular risk stratification in both the general population and those who are at high risk of adverse cardiovascular events [24]. According to the International Federation of Clinical Chemistry and Laboratory Medicine, “high sensitivity” means that an assay for the intended biomarker must be detectable in over 50% of the population who appear to be in good health [25]. For example, a point-of-care testing (POCT) device, such as the HUBI-QUANPro, has been found to have a good comparable analytical performance with a reference laboratory instrument for cardiac troponin I measurement [26]. It is a one-time use, one-step immunoassay tool that uses a reflectance image sensor to measure the amount of cardiac troponin I present in a whole blood or plasma sample. The idea that high-sensitivity cardiac troponin (hs-cTn) assays could be used to stratify cardiovascular risk in the general population is supported by this detectability in asymptomatic individuals.

Nonetheless, clear distinctions between hs-cTnI and hs-cTnT have been noted in their correlations with results. While both hs-cTnI and hs-cTnT were strongly associated with cardiovascular death and heart failure, hs-cTnI was more strongly associated with myocardial infarction and coronary artery disease, and hs-cTnT was more closely associated with non-cardiovascular mortality, according to data from the Generation Scotland Scottish Family Health Study, which followed 19,501 people for 7.8 years [27]. Variations in the genetic loci linked to low-grade cardiac troponin increases, which show little overlap for cTnI and cTnT, could be the cause of this disparity. These results highlight cTnI and cTnT’s potential contribution to improving cardiovascular risk classification by indicating that they offer complementary information rather than redundant data.

#### 2.1.2. High Sensitivity C-Reactive Protein (hs-CRP)

One of the most widely utilized inflammatory biomarkers for determining the risk of atherosclerosis is high-sensitivity C-reactive protein (hs-CRP) [28]. CRP is an acute-phase reactant that is present in blood plasma and rises in reaction to inflammation. In otherwise healthy populations, inflammatory biomarkers, such as hs-CRP, are substantially linked to cardiovascular risk, according to numerous prospective cohort studies [29]. Because of its large clinical data and simplicity of measurement, hs-CRP has emerged as the industry standard biomarker for predicting cardiovascular risk. Its use for risk assessment is also supported by several clinical guidelines, especially for individuals with moderate or atypical cardiovascular risk profiles [30,31,32,33]. The predictive significance of hs-CRP is further supported by recent studies. Early increases in serum hs-CRP are predictive of all-cause mortality as well as the risk of cardiovascular disease (CVD). Furthermore, it has been demonstrated that hs-CRP elevation timing is a major predictor of CVD risk [34]. Another study found that hs-CRP and interleukin-6 (IL-6) have strong discriminative power for evaluating pCAD risk when six biomarkers were added to a traditional risk factor model for predicting pCAD [35]. These results demonstrate how important hs-CRP and other inflammatory biomarkers are for enhancing pCAD diagnosis and early risk assessment.

#### 2.1.3. Brain Natriuretic Peptides (BNP) and N-Terminal Pro-B-Type Natriuretic Peptide (NT-proBNP)

Atrial natriuretic peptide (ANP), C-type natriuretic peptide (CNP), Dendroaspis natriuretic peptide (DNP), and urodilatin are all members of the natriuretic peptide family, which also includes brain natriuretic peptide (BNP), sometimes known as B-type natriuretic peptide. The latter peptides, however, do not yet have any clinical significance [36]. Cardiomyocytes primarily produce BNP in reaction to volume overload or increased ventricular pressure [37]. It functions as a biomarker for left ventricular hypertrophy and dysfunction in addition to myocardial ischemia. BNP is a contender for inclusion in main cardiovascular risk scoring systems because it may enhance the prediction power of well-known biomarkers, such as troponin and C-reactive protein.

According to recent research, even in the absence of irreparable damage or systolic dysfunction, BNP levels may be a good indicator of the degree of ischemia insult and are highly predictive of triple vessel disease [38]. N-terminal pro-B-type natriuretic peptide (NT-proBNP) is a sensitive marker in both acute and chronic heart failure settings and a dependable indication of BNP synthesis, despite its lack of direct clinical usefulness. Because it is non-specific, NT-proBNP can be raised in a number of acute and chronic diseases [39]. Routine BNP or NT-proBNP screening in healthy individuals or for treatment adjustment in diagnosed patients is not advised by current guidelines [40].

### 2.2. Emerging Biomarkers for Premature Coronary Artery Disease

#### 2.2.1. Interleukin 6 (IL-6)

An important player in the pathophysiology of atherosclerosis, interleukin 6 (IL-6) is a multifunctional proinflammatory cytokine with wide-ranging biological effects. IL-6, which is mostly secreted by T cells and macrophages, promotes atheroprogression, plaque instability, and the generation of C-reactive protein (CRP), all of which are factors in the onset and advancement of clinical atherosclerosis [41,42,43]. In numerous investigations, elevated plasma IL-6 levels have been substantially associated with atherosclerotic plaque instability [44,45,46,47], establishing IL-6 as a valid marker for atherosclerosis and coronary artery disease (CAD). Furthermore, atherosclerotic lesions have been demonstrated to express IL-6 at significant levels [48]. Research on animals has shown that IL-6 plays a part in the early aggravation of atherosclerosis; in male mice fed a high-fat and regular diet, recombinant IL-6 treatment was linked to the early formation of atherosclerotic plaques [49].

According to a recent Mexican study, individuals with premature CAD (pCAD) had noticeably greater IL-6 concentrations than healthy controls. The rs1800795 C allele (GC and CC genotypes) was proposed to link with the elevated level of IL-6. IL-6 polymorphisms were also linked to cardiovascular risk variables in pCAD patients, including hypoalphalipoproteinemia and high CRP levels, although not directly linked to pCAD [50]. Furthermore, patients with unstable CAD who had IL-6 levels higher than 5 ng/L had higher 6- and 12-month mortality rates [51]. Greater sensitivity and specificity than hs-CRP in predicting plaque instability were demonstrated by higher IL-6 plasma levels, which were also associated with thin-cap fibroatheroma in patients with ischemic heart disease as seen by optical coherence tomography [52]. These results highlight the importance of IL-6 in predicting the likelihood and severity of pCAD, which makes it a useful biomarker for managing and predicting the course of the disease.

#### 2.2.2. Cell Adhesion Molecules (CAM)

The study of cell adhesion molecules (CAM) as possible biomarkers for the prediction and prevention of coronary artery disease (CAD) began in 1993 when it was discovered that CAM was present in atherosclerotic plaques [53]. Leukocyte adhesion to endothelial cells during acute and chronic inflammation is mediated by CAMs, which belong to the immunoglobulin family [54]. Although their functions in atherosclerotic lesions differ, important molecules, such as E-selectin, soluble intracellular adhesion molecule 1 (sICAM-1), and soluble vascular cell adhesion molecule 1 (sVCAM-1), are involved in lymphocyte migration into the subendothelial region. It has been demonstrated that VCAM-1 is one of these that is especially important in the early and late phases of atherosclerosis [55,56].

In a prospective cohort of patients with existing CAD, the AtheroGene study found that sVCAM-1 was a powerful independent predictor of subsequent fatal cardiovascular events [57,58]. Additionally, a number of studies have shown that in cases with CAD, sVCAM-1 is a more accurate marker than sICAM-1 [15,59,60,61,62]. Its usefulness in the prognostication of CAD is further demonstrated by the positive correlation found between elevated levels of sVCAM-1 and an increased risk of major adverse cardiovascular events (MACE) during a 6-month follow-up [63]. These results highlight sVCAM-1’s potential as a useful biomarker for CAD risk assessment and treatment.

#### 2.2.3. Apolipoproteins

The overall amount of atherogenic and anti-atherogenic particles in the bloodstream can be determined by measuring plasma apolipoproteins. Apoprotein A1 (ApoA1), a significant component of high-density lipoprotein (HDL) involved in reverse cholesterol transport, and Apoprotein B (ApoB), the main apolipoprotein of low-density lipoprotein (LDL) and related lipoproteins, such as very-low-density lipoprotein (VLDL) and intermediate-density lipoprotein (IDL), are both strongly linked to premature coronary artery disease (pCAD) [64,65,66]. The INTERHEART [67] and AFCAPS/TexCAPS [68] investigations have shown a clear linear link between the ApoB/ApoA1 ratio and CAD risk, establishing it as the most reliable predictor of CAD risk.

Reduced ApoA1 and increased ApoB are important predictors of pCAD in hereditary types of dyslipidemia [69]. Their connection to pCAD revealed a gender-specific trend, with ApoB levels having a stronger correlation with pCAD in women and ApoA1 levels being independently linked to pCAD in men [70,71]. These results highlight the significance of ApoA1 and ApoB plasma levels and their ratio in early diagnosis and risk stratification of premature CAD by indicating that they are better predictors of pCAD risk than conventional plasma lipoprotein measures.

#### 2.2.4. CC Chemokines

CC cytokines are essential in controlling leukocyte migration and activation in the heart and major arteries. The CC chemokine RANTES (Regulated upon Activation, Normal T Cell Expressed and Secreted) is expressed by a variety of cells, including T cells, fibroblasts, and certain monocytes, and it has a role in the pathophysiology of cardiovascular disease [72,73,74]. By promoting leukocyte chemotaxis and trans-endothelial migration along the endothelium, RANTES contributes to the early phases of atherogenesis. RANTES, which is stored in platelet α-granules, is released onto the surface of damaged endothelial cells during platelet activation. This causes monocyte recruitment and accelerates the development of atherosclerotic plaque [73,74]. This process demonstrates its important function in promoting atherogenesis by means of interactions between immune cells and activated platelets.

Similarly, another CC chemokine linked to cardiovascular disease is CCL2, which is involved in attracting and activating T cells, NK cells, and monocytes/macrophages at inflammatory areas [75,76,77,78]. In animal models, CCL2 promotes the development of atherosclerotic plaque, especially in mice lacking apolipoprotein E, whereas mice lacking CCL2 have fewer macrophages in the aorta [75,76]. With elevated RANTES levels associated with high-risk groups in early plaque formation in patients with stable angina, recent studies on gene polymorphisms of the chemokine RANTES/CCL5 have shown their potential as predictive risk factors for coronary artery disease [79,80,81,82]. Furthermore, patients with acute myocardial infarction and stable angina had around three times better circulating RANTES concentrations than controls, although CCL2 levels were only slightly higher [83]. While both RANTES and CCL2 are markers for atherosclerosis rather than its severity, these results imply that RANTES is a more reliable biomarker for identifying early atherosclerotic lesions.

#### 2.2.5. Adiponectin

Adiponectin is a protein derived from adipocytes that is crucial for regulating insulin sensitivity. Various studies have indicated that adiponectin plays a significant part in atherosclerosis and may be linked to coronary artery disease (CAD). Plasma adiponectin was quantified through an ELISA test in 568 individuals of French-Canadian origin with familial hypercholesterolemia who were not diabetic. CAD manifests at a considerably younger age in familial hypercholesterolemia patients in the lowest tertile of plasma adiponectin [84]. These findings imply that, in addition to the already heightened risk observed in familial hypercholesterolemia patients, decreased plasma adiponectin is linked to an elevated risk of premature CAD.

Another study observed a significant association between single nucleotide polymorphisms (SNPs) (+276 G>T) of the adiponectin gene and CAD. Moreover, this SNP was found to be a significant predictor of lower levels of serum adiponectin in CAD subjects with early-onset disease [85]. Based on these findings, the adiponectin gene variant, or a mutation in linkage with it, determines lower levels of adiponectin protein expression, which in turn increases the risk of developing insulin resistance, atherosclerosis, and cardiovascular disease. Given that other risk factors of CAD are less common in younger individuals than in older age groups, genetic variables for late-onset illnesses may have a stronger effect in those with early-onset CAD due to the significant association of the adiponectin gene.

#### 2.2.6. Homocysteine

Homocysteine is an amino acid that can be metabolized via the trans-sulfuration pathway to cysteine or the remethylation pathway to methionine. Both pathways rely on several other biochemical enzymes, including folic acid and vitamin B12, as well as enzymes, such as methionine synthetase (MS) and methylenetetrahydrofolate reductase (MTHFR). Pyridoxine (vitamin B6) and the enzyme cystathionine beta synthetase (CBS) are required for a subsequent process. Hyperhomocysteinemia can be caused by a nutritional shortage, a loss of one of those mechanisms, or both. Indeed, epidemiological research has shown that increased circulating homocysteine due to endothelial dysfunction, oxidative stress, and enhanced thrombogenicity is associated with the advancement of atherosclerotic plaques [86,87].

Hyperhomocysteinemia is an independent risk factor for CAD in patients younger than 45 years old, especially men. However, there was no significant association in women. A study showed that in men, there was a 2.4 times higher risk of premature CAD with homocysteine plasma levels of more than 15 μmol/L [88]. Similarly, many studies have shown that elevated total homocysteine concentration is an independent risk factor for pCAD [89,90,91,92].

#### 2.2.7. Tumour Necrosis Factor-Alpha (TNF-a)

TNF-a, also known as tumor necrosis factor-alpha, is a crucial risk factor for the development and course of atherosclerosis [93]. The risk of pCAD may be influenced by polymorphisms in cytokine genes [94], and several molecular networks are connected to the pathogenesis of pCAD [95]. TNF-a, sometimes referred to as the transducer of cardiovascular disorders, specifically pCAD, controls the expression of adhesion molecules and cytokine networks as well as several signal transduction pathways [96,97]. Studies have shown that TNF-a and TNF-a receptor polymorphisms have been linked to CAD and ischemic heart disease [98,99,100,101]. A case-control study among 98 pCAD Pakistani patients showed that TNF-a was significantly raised in pCAD patients (*p*  <  0.01), and the degree of angiographic blockage was significantly linked with TNF-a (*p* < 0.01) [102]. This suggests pro-inflammatory cytokines, such as TNF-a, can be used to determine the extent of atherosclerosis and are important in the pathophysiology of pCAD.

## 3. Application of Biosensors for the Detection of Premature Coronary Artery Disease

A biosensor is defined by the International Union of Pure and Applied Chemistry (IUPAC) as an independent, integrated device that may gather data by making direct spatial contact between a biological recognition component (biochemical receptor) and a transduction element [103]. When obtaining analytical data, IUPAC also highlights the need to differentiate biosensors from more comprehensive bioanalytical systems [103]. The development of biosensors has accelerated in recent years, especially for applications in health and the environment where biomarkers are used for predictive or diagnostic purposes. Recent reviews have collated the list of biosensors development for cardiac biomarkers [19,104]. Membrane receptors, oligonucleotides, aptamers, enzymes, and whole cells are examples of biochemical receptors in biosensors. Similar to their use in ELISA assays, particular antibodies are most frequently used for the detection of cardiac biomarkers, such as troponin, D-dimers, and BNP. A variety of transduction approaches are then used to transform the biorecognition event into a quantifiable physical signal [105].

Common transduction techniques include mechanical techniques, such as detecting mass changes on mechanical resonators, as in quartz microbalance systems; optical techniques, such as surface plasmon resonance setups that measure changes in the refractive index of the bioactive layer upon biomarker binding; and electrical techniques, such as measuring the electrical charges of biomarkers with transistors or nanowires. Although many transduction pathways have been investigated for the detection of cardiac biomarkers, electrochemical and optical techniques continue to be the most popular and thoroughly studied. Utilizing micro- and nanotechnology, biosensors facilitate point-of-care testing at the patient’s bedside by facilitating simple prototyping and portability. However, even though several biosensors are supplied as benchtop devices, not all of them are portable. Since biosensors have the potential to achieve lower detection thresholds and greater accessibility than established gold-standard procedures, including ELISA assays conducted on automated central laboratory platforms, they present interesting alternatives [106].

### 3.1. Electrochemical Biosensors

Premature coronary artery disease (pCAD) can be detected early and managed at the point of care through electrochemical biosensors, especially those that use Electrochemical Impedance Spectroscopy (EIS). EIS is a crucial electrochemical method for measuring circuit impedance. It exhibits numerous benefits, including steady-state operation, small-signal analysis, and the capacity to examine signal relaxations throughout a broad frequency range. The ease of use, portability, affordability, and disposability of electrochemical biosensors make them well suited for point-of-care applications. To enable electrochemical sensing, these biosensors usually use a three-electrode electrochemical cell, which consists of a working electrode, a counter electrode (CE), and a reference electrode. EIS is very useful for studying interfacial characteristics during specific bio-recognition processes, including whole-cell recognition, protein recognition, receptor identification, nucleic acid detection, and antigen–antibody interactions. The creation of aptasensors and immunosensors has, thus, been the subject of numerous studies on EIS-based biosensors [107].

In contrast to other electrochemical methods, EIS provides a wealth of information by distinguishing between diverse electrical, electrochemical, and physical processes that take place in a complicated electrochemical system [108]. Biological sample analysis is made quick, easy, and extremely sensitive with EIS-based detection techniques. In routine cardiac examinations, for example, the measurement of cardiac troponin I (cTnI) is essential both before and during the start of symptoms of acute myocardial infarction (AMI). This emphasizes the necessity of an easy-to-use, reliable, and accurate label-free EIS detection technique that can track cTnI trace levels, enabling effective therapeutic management and early diagnosis of AMI and associated disorders [109].

Table 1 presents a comprehensive overview of various electrode modification approaches as well as electrochemical biosensors and their performance metrics for detecting biomarkers associated with premature coronary artery disease (pCAD). Electrochemical biosensing techniques have not been established for all cardiac biomarkers. Nevertheless, we include biomarkers for electrochemical biosensing techniques that have not yet been discovered in this review in order to provide a wide biomarker range and to address the gaps in the current biosensor research. Out of the 15 biomarkers for premature coronary artery disease, 14 biomarkers were detected via electrochemical biosensing techniques. The most common electrochemical biosensing technique used was Electrochemical Impedance Spectroscopy (EIS). Human serum was the commonest sample used followed by whole blood, spiked serum, bovine serum albumin (BSA), saliva and urine.

Cardiac troponin I (cTnI) and cardiac troponin T (cTnT) are two key biomarkers used in the diagnosis of pCAD. While both are troponins, they have distinct properties that can affect their detection in electrochemical biosensing techniques. Table 1 summarizes various electrochemical biosensors for detecting cardiac troponin I (cTnI) and cardiac troponin T (cTnT), detailing their linear detection ranges, limits of detection (LOD), and the types of samples used. For cTnI, sensors employ techniques, such as Differential Pulse Voltammetry (DPV), Cyclic Voltammetry (CV), and Electrochemical Impedance Spectroscopy (EIS), with detection ranges spanning from 10 fg/mL to 1000 ng/mL and LODs as low as 0.0005 ng/mL, predominantly using human serum. These sensors use diverse materials, including carbon-based electrodes, gold nanoparticles, and various nanocomposites. For cTnT, methods include DPV, CV, and EIS, with detection ranges from 0.02 ng/mL to 10 µg/mL and LODs as low as 0.008 ng/mL, applied to human serum and blood plasma. Biosensors for cTnI are typically designed to achieve very low limits of detection (LOD) due to their rapid release and critical role in early diagnosis. While also achieving low LODs, the biosensors for cTnT must account for potential cross-reactivity with non-cardiac troponins. Special adjustments in the sensor design and functionalization are crucial to enhance specificity and reduce false positives.

The biosensor designed for detecting Interleukin 6 (IL-6) utilizes gold nanoparticles (AuNPs) on a screen-printed electrode (SPE) combined with Differential Pulse Voltammetry (DPV) for highly sensitive detection. This setup achieves a detection range from 10^2^ to 10^8^ femtomolar (fM), with an impressive limit of detection at 47.9 fM, indicating the capability to detect IL-6 at very low concentrations. The use of AuNPs enhances the sensor’s sensitivity by increasing the effective surface area and improving the electrochemical response, making it a powerful tool for monitoring IL-6 in biological samples. AuE (Gold Electrode) biosensor is a type of electrochemical biosensor that utilizes gold electrodes for detecting various biomarkers, including Tumor Necrosis Factor-alpha (TNF-α). AuE with Alternating Current Voltammetry (ACV) provides the widest detection range, whereas Gamma-Propyltriethoxysilane-ITO-Polyethylene Terephthalate (GPTES-ITO-PET) sensor, using detection techniques, such as CV and EIS, offers the lowest LOD.

Electrochemical biosensing techniques for BNP generally offer a wide range of detection capabilities, with LODs ranging from 3.34 fg/mL to 4 pg/mL. These sensors are optimized for human serum. NT-proBNP sensors often provide lower LODs, particularly those using Amperometry and EIS, with limits as low as 0.02 pg/mL, reflecting a higher sensitivity compared to BNP sensors. NT-proBNP sensors can be used for both human serum and saliva. While both BNP and NT-proBNP sensors use electrochemical techniques to detect peptides, NT-proBNP sensors typically achieve lower detection limits and higher sensitivity and are designed for a broader range of sample types compared to BNP sensors.

The detection of low-density lipoprotein (LDL) via anti-apolipoprotein B 100 and apolipoprotein A1 using electrochemical biosensing techniques differs in several key aspects. Electrochemical biosensing techniques for the detection of LDL often focus on methods, such as Electrochemical Impedance Spectroscopy (EIS), Differential Pulse Voltammetry (DPV), and Linear Sweep Voltammetry (LSV), for a wider concentration range and higher sensitivity, while those for apolipoprotein A1 emphasize techniques, such as EIS and Square Wave Voltammetry (SWV), providing greater sensitivity for detecting very low concentrations.

**Table 1:** Electrochemical biosensing techniques with their linear ranges and limit of detection (LOD) of pCAD-associated biomarkers.

Biomarkers for pCAD	Sensor	Technique	Linear Range	LOD	Relative Standard Deviation (%)	Sample Type	Recovery	Study Reference
Cardiac Troponin I (cTnI)	N, Zn-GQDs/GCE	DPV	10–10^6^ pg/mL	4.59 pg/L	9.09–11.1	Human serum	92–97.1	[110]
	COOH-ZnONPs/GCE	EIS DPV	1.25 × 10^5^–8.25 μg/mL	2.61 × 10^5^ μg/mL	3.06–4.5	Human serum	93.40–114.28	[111]
	CSA/MCH/Fc-COFNs-MBA/Au	CV DPV	10 fg/mL–10 ng/mL	2.6 fg/mL	4.2	Human serum	97.2–102.9	[112]
	PCN-AuNPs/LSGE	CV SWV	0.0001–1000 ng/mL	0.01 pg/mL	2.25	Human serum	NR	[113]
	pCTAB/DES/Au-SPE and pCTAB/DES/Ab2/Au-SPE	DPV CV	0.04–50 ng/mL	0.0009 ng/mL	0.37–1.94	Human serum	NR	[114]
	N-prGO/COOH/PEG-aptamer/GCE	DPV	0.001–100 pg/L	1 pg/mL	4.3	Human serum	98.2–101.7	[115]
	Fc-COOH-CIL-HCNTs/GCE	DPV	0.01–60 ng/mL	0.006 ng/mL	4.3–6.0	Human serum	96.4–103.3	[116]
	Ti disc/AuNPs/Apt	DPV	1–1100 pM	0.18 pM	3.28	Human serum	100.2–101.8	[117]
	DNA 3WJ/MB/Apt	CV	0 pM–100 nM	1.0 pM	NR	Human serum	NR	[118]
	MIP/BNQDs/GCE	DPV	0.01–5.0 ng/mL	0.0005 ng/mL	0.17–0.47	Human plasma	NR	[119]
Cardiac Troponin T (cTnT)	N-MIP/SPCE	DPV	0.02–0.09 ng/mL	0.008 ng/mL	NR	Human serum	NR	[120]
	cTnT-PANI/PMB/f-MWCNTs/ SPCE	DPV CV	0.10–8.0 pg/mL	0.040 pg/mL	1.3	Human blood plasma	91–112	[121]
	GCE	CV DPV	10 pg/mL–10 µg/mL	0.44 pg/mL	5.93–12.02	Human serum	NR	[122]
	BSA/ZnO/MPC/IL/anti-CRP/CPE	EIS DPV	0.01–1000 ng/mL	0.005 ng/mL	<6	Human serum	94.5–107.0	[123]
	PMPC-SH/SAM/AuNPs/SPCE	DPV	5–5000 ng/mL	1.6 ng/mL	<1.34	Human serum	NR	[124]
	PEI-Fc /anti-CRP/GCE	DPV EIS	10–50,000 ng/mL	0.5 ng/mL	8.5	Blood sample	NR	[125]
	MB-NH2 -SWCNT-AuNPs/SPE	CV DPV EIS	5 pg/mL–1 µg/mL	5 pg/mL	>13.38	Blood sample	80	[126]
	Fc-ECG/MEL/AuNPs/SPE	CV EIS DPV	0.001–1000 µg/mL	0.30 µg/mL	6.59	Human serum	98.69–102.43	[127]
	anti-CRP rGO/Ni/PtN/SPCE	Amperometry	2–100 µg/mL	0.80 µg/mL	8.0	Human serum	NR	[128]
	MWCNTs/AuE	EIS CV	0.084–0.84 nM	4\0 pM	3.15	Human serum	NR	[129]
	ERGO/PTyr	DPV EIS	1.09–100 µg/L	0.375 µg/L	NR	Human serum	NR	[130]
	BSA/anti-CRP/MPA/Au	CV SWV	5–220 fg/mL	2.25 fg/mL	3.12	Human serum	NR	[131]
Interleukin 6 (IL-6)	AuNP-SPE	DPV	10^2^–10^8^ fM	47.9 fM	NR	Human DNA	NR	[132]
Tumour necrosis factor- alpha (TNF-a)	Anti-TNFα/BSA/PAMAM/ NFs-AuE	CV EIS	10–200 pg/mL	669 fg/mL	NR	Human serum Saliva	NR	[133]
	AuE	ACV	0.1–500 nM	100 pM	NR	Urine Saliva	NR	[134]
	TNFα/anti-TNFα-Ab1/AuNPs/S-MWCNTs/GCE	CV EIS	0.01–1.0 pg/mL	2.00 fg/mL	0.61	Human plasma	100	[135]
	AuHCF-AuNPs/SPE	DPV	10 pg/mL–40 µg/mL	5.5 pg/mL	0.46	Human serum	NR	[136]
	ITO Electrode	EIS CV	0.02–4 pg/mL	6 fg/mL	NR	Human serum	97.07–100.19	[137]
	PDMS/AuE- ITO	CV	0.15 pg/mL–15 ng/mL	0.07 pg/mL	NR	Human serum	NR	[138]
	GPTES-ITO-PET	CV EIS	0.01–1.5 pg/mL	3.1 fg/mL	0.87	Human serum	96.51–100.90	[139]
	AuE (microelectrodes)	CV EIS	1–15 pg/mL	NR	NR	Human saliva	NR	[140]
Brain natriuretic peptides (BNP)	AuNPs-S-Phe/SPCE	EIS CV	0.014–15 ng/mL	4 pg/mL	6.4	Human serum	NR	[141]
	PPIX/N–ZnO NP/ITO	EIS	1 pg/mL–0.1 µg/mL	0.14 pg/mL	2.6–5.9	Human serum	90.0–102	[142]
	ZnCo_2_O_4_/N-CNTs-Ab/GCE	Amperometry DPV CV	0.01 pg/mL–1 ng/mL	3.34 fg/mL	2.9–3.5	Human serum	97.0–102.1	[143]
N-terminal pro-B-type natriuretic peptide (NT-proBNP)	Au@PdPtRTNs/GCE	Amperometry CV EIS	0.1 pg/mL–100 ng/mL	0.046 pg/mL	3–5.4	Human serum	98.7–101.3	[144]
	SPE, Pt counter electrode	EIS	0.02–1 pg/mL	0.02 pg/mL	NR	Saliva	99 ± 8	[145]
	Paper Electrode	LASV SWASV	53–590 pM	300.0 pM	NR	Human serum	NR	[146]
Adiponectin	3-GOPS/anti-adiponectin-ITO-PET	EIS CV	25–2500 pg/mL	148 pg/mL	NR	Human serum	NR	[147]
	GP	EIS CV	0.05–25 pg mL^−1^	0.0033 pg mL^−1^	NR	Human serum	NR	[148]
	MIP/GWE	EIS CV	0–50 μg mL^−1^	0.25 μg mL^−1^	NR	Human serum	NR	[149]
Low density lipoprotein (LDL) via Anti-apolipoprotein B 100	AuNPs-AgCl@PANI-mGCE	EIS	3.4–134 ng/dL	3.4 ng/dL	1.9	BSA	NR	[150]
	NH_2_-Rgo/ITO–CGE	EIS	5–120 mg/dL	5 mg/dL	NR	Human serum	NR	[151]
	CNT-CH/ITO-CGE	EIS	0–120 mg/dL	12.5 mg/dL	NR	BSA	NR	[152]
	NiO/ITO/glass bioelectrode	DPV CV EIS	0.018–0.5 μM	0.015 μM	NR	BSA	NR	[153]
	Fe_3_O_4_@SiO_2_ and MOF-Fc@aptame	SWV	1.0 ng·mL^−1^–100 μg·mL^−1^	0.3 ng/mL	NR	Human serum	NR	[154]
	Au/4-ATP/AbM/BSA	SWV	0.01 to 1.0 ng/mL	0.31 ng/mL	NR	Human serum	NR	[155]
	Au/Apt	SWV	0.01 and 1.0 ng/mL	0.25 ng/mL	NR	Human serum	NR	
Low density lipoprotein (LDL) via Cholesterol esterase/Cholesterol oxidase/Anti-apolipoprotein B 100	GCE/poly(oATP)/AuNP	LSV	10–1000 ng/mL	3.25 ng/mL	NR	Human serum	NR	[156]
Apolipoprotein A1	Functionalized gold nanoparticles protein (FAuNP) composite films	EIS	0.1–10 ng mL^−1^	50 pg mL^−1^	NR	Human serum	NR	[157]
	AuNR/AuNW/CS nanocomposite electrochemical aptasensor	CV DPV	0.1 to 1000 pg mL^−1^	0.04 pg mL^−1^	NR	Spiked serum	NR	[158]
Homocysteine	MIP-modified nanocomposite screen-printed carbon electrode	Voltammetry	5.0–150 µM	1.2 µM	NR	Human serum	91.10–95.83	[159]
	Aptamer-modified Au NP/graphene sponge electrode	Voltammetry	1–100 µM	1.0 µM	NR	BSA	NR	[160]
	Aptamer-modified gold nanoparticle/carbon electrode	DPV	0.05–20 µM	0.009 µM	NR	Human serum Urine	NR	[161]
CC Chemokines: CCL5/RANTES (Regulated upon Activation, Normal T cell Expressed and presumably Secreted)	Sandwich type immunosensor with neutravidin-functionalized magnetic microparticles (Neu-MBs) modified with a biotinylated antibody (anti-CCL5-Biotin)	Amperometry	0.1–300 ng·mL^−1^	40 pg/mL	NR	Human serum	NR	[162]
CC Chemokines: CCL2/MCP-1 (monocyte chemoattractant protein 1)	Sandwich-type electrochemical immunosensor with cAb immobilized on rGO-(rGO-TEPAThi-Au)/GCE	Amperometry	20 fg/mL–1000 pg/mL	8.9 fg/mL	NR	Spiked serum	NR	[163]
	Label-free electrochemical immuno-sensor with cAb immobilized on Au@Pt-CA-AuE	DPV	0.09–360 pg/mL	0.03 pg/mL	NR	Spiked serum	NR	[164]
	Sandwich-type immunosensor with cAb immobilized onto a silicon photonic micro ring resonator	Res λ shift	84.3–1582.1 pg/mL	0.5 pg/mL	NR	Spiked serum	NR	[165]
	Label-free electrochemical immunosensor with cAb immobilized on PtNPs/SWCNHs	Amperometry	0.06–450 pg/mL	0.02 pg/mL	NR	Human serum	NR	[166]
Cell adhesion molecules (CAM): Soluble vascular cell adhesion molecule 1 (sVCAM-1)	Sandwich immunoassay with thiol-conjugated gold microelectrodes	EIS CV	8 fg/mL–800 pg/mL	8 fg/mL	NR	Urine	NR	[167]
Multitarget antibody (cTnI, CRP)	TiO_2_ nanofibrous	ELISA	cTnI: 10 pg/mL–100 ng/mL CRP: 1 pg/mL–100 ng/mL	cTnI: 37 pg/mL CRP: 0.8 pg/mL	NR	Whole blood	NR	[168]
Multitarget aptamer (Myoglobin, cTnI)	HsGDY@NDs	EIS	Myoglobin: 10 fg/mL–1 ng/mL cTnI: 10 fg/mL–100 ng/mL	Myoglobin: 9.04 fg/mL cTnI: 6.29 fg/mL	NR	Human serum	NR	[169]
Cell adhesion molecules (CAM): Soluble intracellular adhesion molecule 1 (sICAM-1)	NR	NR	NR	NR	NR	NR	NR	NR

NR: not reported.

Electrochemical biosensors for detecting adiponectin include the 3-Glycidoxypropyltrimethoxysilane (3-GOPS)/anti-adiponectin-ITO-PET sensor, which utilizes Indium Tin Oxide (ITO) and Polyethylene Terephthalate (PET) for its electrode system and employs EIS and CV for comprehensive electrochemical analysis. This biosensor operates within a detection range of 25 pg/mL to 2500 pg/mL and a limit of detection (LOD) of 148 pg/mL, suitable for human serum. The graphite paper (GP) sensor also employs EIS and CV, achieving a broader linear range of 0.05–25 pg/mL and an impressive LOD of 0.0033 pg/mL, making it highly sensitive to human serum.

An electrochemical biosensor for homocysteine detection typically involves a combination of a specific electrode modification and an electrochemical technique to achieve high sensitivity and selectivity. Molecularly imprinted polymers (MIPs) are used to create selective binding sites for homocysteine. These sensors can detect homocysteine in a range of 5.0–150 µM with a limit of detection (LOD) of 1.2 µM, often used in human serum. Aptamers are oligonucleotides that bind specifically to homocysteine. Sensors using aptamer modifications, such as gold nanoparticles (AuNP) combined with graphene sponges or carbon electrodes, showed a range of detection from 1 to 100 µM with an LOD of 1.0 µM. Some advanced aptamer-based sensors achieve very low detection limits, such as 0.009 µM, and can be used with both human serum and urine.

In the context of CC chemokines, electrochemical biosensing techniques for CCL5/RANTES generally focus on amperometric methods with moderate sensitivity, while those for CCL2/MCP-1 include a broader range of advanced techniques, such as DPV and optical resonance, with higher sensitivity and a broader detection capability. Table 1 also describes an electrochemical biosensor for detecting soluble vascular cell adhesion molecule 1 (sVCAM-1), utilizing a sandwich immunoassay approach with thiol-conjugated gold microelectrodes. This sensor employs Electrochemical Impedance Spectroscopy (EIS) and Cyclic Voltammetry (CV) techniques, offering a detection range from 8 fg/mL to 800 pg/mL, with a limit of detection (LOD) as low as 8 fg/mL. The sensor is designed for use with urine samples.

Overall, the data highlight the diverse capabilities of modern biosensors in the precise detection and quantification of pCAD biomarkers across different sample types. There were no data on electrochemical biosensors for soluble intracellular adhesion molecule 1 (sICAM-1).

In our review, we have also identified two studies that discussed multiplexed biosensing techniques for pCAD biomarkers. Table 2 compares two different multiplexed biosensor technologies for detecting biomarkers related to premature coronary artery disease. The first sensor uses multi-target antibodies with titanium dioxide (TiO_2_) nanofibrous material and ELISA (Enzyme-Linked Immunosorbent Assay), offering detection ranges of 10 pg/mL to 100 ng/mL for cTnI and 1 pg/mL to 100 ng/mL for CRP, with detection limits of 37 pg/mL for cTnI and 0.8 pg/mL for CRP, and is suitable for whole blood samples [168]. The second sensor employs multitarget aptamers using hetero-nanostructures of nanodiamonds (NDs) and hydrogen-substituted graphdiyne (HsGDY) (denoted as HsGDY@NDs) with EIS (Electrochemical Impedance Spectroscopy), providing lower detection limits of 9.04 fg/mL for myoglobin and 6.29 fg/mL for cTnI, with detection ranges of 10 fg/mL to 1 ng/mL for myoglobin and 10 fg/mL to 100 ng/mL for cTnI, and is designed for human serum samples [169]. This comparison highlights the differences in technology, sensitivity, and sample types used in these biosensor systems. Sandwich-type aptamer-based biosensors are a subset of biosensors that utilize two aptamers in combination to detect a target molecule (often a pathogen, biomarker, or tiny chemical) with high sensitivity and specificity. A variety of detection techniques, including electrochemical, surface plasmon resonance (SPR), optical, and colorimetric platforms, have been used to construct sandwich-type aptamer-based biosensors. In a recent study, electrochemical aptasensors exhibited a lower detection limit (LOD) than colorimetric transduction and antibody or enzyme techniques [170]. Based on spectrophotometric experiments, the novel hexaferrocenium tri[hexa(isothiocyanato)iron(III)]trihydroxonium (HexaFc) complex has shown high DNA binding and selectivity. It was then effectively employed as a novel redox indication for an electrochemical DNA biosensor [171].

Multiplexed biosensors designed to detect biomarkers of premature coronary artery disease face several challenges that can impact their effectiveness and adoption. One primary challenge is achieving high sensitivity and specificity for each target biomarker within a single device, as cross-reactivity or interference among biomarkers can lead to inaccurate results. Additionally, the complexity of integrating multiple detection technologies and ensuring reliable performance across different biomarkers can be technically demanding, requiring advanced materials and precise calibration. Sample variability, such as differences in blood composition, can also affect sensor accuracy and reproducibility. Moreover, the cost and complexity of developing and manufacturing these sophisticated devices can limit their widespread use. Finally, data interpretation from multiplexed readings necessitate advanced algorithms and robust analytical frameworks to provide clear and actionable insights for early disease detection and management.

### 3.2. Clinical Validation and Real-World Applications Electrochemical Biosensors

Recent research has underscored the clinical validation and practical uses of electrochemical biosensors for identifying pCAD. A study from 2021 presented a detection algorithm utilizing an electrochemical biosensor for acute coronary syndrome, reaching high specificity (94%) and sensitivity (92%) through the analysis of troponin levels, indicating its potential for at-home cardiovascular monitoring [172]. Progress in electrochemical biosensors was highlighted in a later study, emphasizing novel electrode designs, signal amplification methods, and microfluidic systems that improve the identification of cardiovascular biomarkers, thus showcasing their practical uses in clinical diagnostics [173]. Moreover, another literature has emphasized the importance of electrochemical immunosensors in point-of-care cardiac biomarker identification, stressing their capability to deliver fast, sensitive, and affordable diagnostic options for early pCAD detection and tailored treatment [174]. These investigations altogether have shown the promise of electrochemical biosensors in enhancing the early detection and observation of pCAD.

### 3.3. Future Prospects and Research Directions

Recent developments in electrochemical biosensors for the identification and tracking of biomarkers linked to premature coronary artery disease (pCAD) are highlighted in this review. The development of quick, sensitive, and accurate biosensors is essential for early diagnosis, which can save lives and subsequently lower medical expenses. This review is novel since it suggests using it as a basis for creating multiplexed biosensors that can identify pCAD biomarkers at the point of care. A future biosensor-based device should prioritize price, usability, and accessibility. This should include a complete package of multiple features, including low-cost signal readouts, visual or smartphone-assisted detection, and simplified result interpretation for novice users. These devices should be leveraged as one of the risk assessment and diagnostic tools and be accessible to the end users. By overcoming these obstacles, electrochemical biosensors will be able to be incorporated into standard clinical diagnostic procedures.

To increase their effectiveness and clinical relevance, future studies on electrochemical biosensors for pCAD biomarker detection should concentrate on a few key aspects. A future biosensor device should have multiple biomarker detection with cutting-edge materials and should have a high yield of biomarker sensitivity and specificity. With the current technology, biosensors integrated with digital devices, such as smartphones, for easy data interpretation and real-time monitoring will gather more big data for further risk stratification in a large population. Thus, efforts should also be made to streamline the production of biosensors for scalability and affordability to ensure their usability within places with limited resources. Electrochemical biosensors may become essential instruments in regular clinical diagnosis and preventive cardiology if these logistical and technical obstacles are resolved.

To enhance the specificity of multiplex biosensors for identifying pCAD, highly selective recognition components, such as monoclonal antibodies, aptamers, or nanomaterials, can be employed to focus on biomarkers, such as CRP and oxidized LDL, with minimal cross-reactivity. Techniques for surface functionalization, such as molecular imprinting or self-assembled monolayers, can be utilized to improve the specificity of sensors even more. Biosensors designed to identify pCAD can be combined with artificial intelligence (AI)-driven diagnostics for continuous monitoring by merging the biosensors’ capacity to identify biomarkers with AI techniques that analyze the information in real time. The biosensors would gather real-time data from patients, which would then be analyzed by machine learning models to detect early indicators of CAD. For example, a slight increase in biomarkers can signify inflammation or endothelial dysfunction. AI algorithms can track trends over time, sending alerts for notable changes or new patterns that need attention. To ease implementation, partnerships with hospitals and diagnostic firms are essential. Hospitals can offer practical clinical settings for examining and confirming these biosensors, whereas diagnostic firms can assist in increasing production and ensuring regulatory adherence for commercial application. Collectively, networks can be created for data exchange and integration with electronic health records (EHR), allowing seamless data transfer between biosensors and patient care systems. Collaborations would also guarantee that essential clinical trials, patient education, and regulatory approvals are obtained, fostering the integration of AI-powered biosensors into conventional healthcare practices for pCAD detection and continuous monitoring.

### 3.4. Limitations of Conventional Diagnostic Tools in the Detection of Premature Coronary Artery Disease

Traditional diagnostic tools for premature coronary artery disease (pCAD) encounter various challenges that impede timely and precise identification. Conventional techniques, such as coronary angiography and stress tests, are commonly employed to identify CAD, but they usually reveal the condition only after considerable plaque accumulation or other symptoms have arisen. These techniques are intrusive, costly, and not suitable for examining younger people, particularly considering the distinct risk factors linked to pCAD. Furthermore, traditional tools frequently depend on a single biomarker or risk factor, failing to offer a complete evaluation of the disease in younger patients who might exhibit uncommon risk factors.

Additionally, conventional diagnostics, such as electrocardiograms (ECGs) and stress tests, frequently do not possess the sensitivity and specificity required for the early detection of pCAD, since these assessments may fail to indicate early-stage atherosclerotic alterations in younger individuals. Consequently, pCAD may go undetected until more serious symptoms or complications arise. Dependence on expensive, invasive techniques, such as coronary angiography, renders extensive screening unfeasible, particularly when more effective options could be utilized. The drawbacks of these traditional methods have prompted the advancement of innovative diagnostic technologies, such as multiplex biosensors, that are better adapted for the early identification and risk evaluation of pCAD in younger groups.

### 3.5. Advantages and Limitations of Electrochemical Biosensors for the Detection of Premature Coronary Artery Disease

Electrochemical biosensors provide notable benefits for identifying pCAD, especially regarding swift, real-time diagnostic capabilities. These sensors are small, easy to transport, and ideal for point-of-care uses, allowing for fast and straightforward screening, even in distant or resource-poor environments. Electrochemical biosensors, known for their high sensitivity and specificity, can identify specific biomarkers linked to pCAD, facilitating early disease detection, frequently before symptoms manifest. Their capacity to identify several biomarkers at once (multiplexing) improves diagnostic precision, offering a thorough perspective on cardiovascular well-being. Moreover, electrochemical biosensors are economical in comparison to conventional diagnostic techniques, such as coronary angiography, rendering them an appealing choice for extensive screening and early diagnosis.

Nonetheless, electrochemical biosensors possess certain drawbacks. The accessibility and dependability of biomarkers for pCAD continue to develop, and existing sensors might not detect the complete range of biomarkers needed for precise diagnosis. Interference from other substances in biological samples can also impact their accuracy. Calibration and standardization challenges can affect the consistency of outcomes, while differences in sample quality or sensor effectiveness may obstruct reliability. Moreover, although these biosensors are user-friendly, their creation and production demand advanced technology and specialized knowledge, potentially restricting their broad implementation in various environments. Despite these obstacles, electrochemical biosensors show significant potential for enhancing the early identification and treatment of pCAD as the technology advances.

## 4. Conclusions

Premature coronary artery disease (pCAD) presents a significant challenge for identification, prediction, and diagnosis. As a result, biomarker evaluation is crucial for effective disease management. This review focuses on innovative approaches in electrochemical biosensors for detecting pCAD biomarkers, highlighting recent advancements in the field. Distinct from traditional coronary artery disease (CAD) biomarkers, novel pCAD biomarkers have been identified, with 14 key biomarkers examined through electrochemical biosensing methods. These sensors offer benefits, such as early detection and intervention capabilities at the point-of-care, due to their potential for miniaturization into wearable and implantable devices, cost effectiveness, wide linear ranges, and low limits of detection (LOD). To enhance the performance of these biosensors, including their selectivity and sensitivity, hybrid systems incorporating various materials, particularly nanomaterials, have been employed. Electrochemical impedance spectroscopy (EIS) emerged as the most commonly used technique, with the majority of samples being biological, such as human serum. Overall, researchers are focused on developing sensors that will provide significant advantages for clinical and point-of-care applications.

## Figures and Tables

**Table 2:** Multiplexed biosensor technologies for detecting biomarkers related to premature coronary artery disease.

Multiplexed Biomarkers for pCAD	Sensor	Technique	Linear Range	LOD	Relative Standard Deviation (%)	Sample Type	Recovery	Study Reference
Multitarget antibody (cTnI, CRP)	TiO_2_ nanofibrous	ELISA	cTnI: 10 pg/mL∼100 ng/mL CRP: 1 pg/mL∼100 ng/mL	cTnI: 37 pg/mL CRP: 0.8 pg/mL	NR	Whole blood	NR	[168]
Multitarget aptamer (Myoglobin, cTnI)	HsGDY@NDs	EIS	Myoglobin: 10 fg/mL∼1 ng/mL cTnI: 10 fg/mL∼100 ng/mL	Myoglobin: 9.04 fg/mL cTnI: 6.29 fg/mL	NR	Human serum	NR	[169]

NR: not reported.

## Data Availability

The data presented in this study are available on request from the corresponding author.

## Abbreviations

The following abbreviations are used in this manuscript:

AuNPs	gold nanoparticles
ACV	alternating current voltammetry
AuE	gold electrode
ASPE	poly-anthranilic acid
AuHCF	layers of gold hexacyanoferrate
AgNp	silver nanoparticle
Ab	antibody-BNP
Ab2	anti-CTnI polyclonal antibody
Au/Fc@CuxO SPs	Rocene-functionalized cuprous oxide superparticles
Apt	aptamer
ASV	anodic stripping voltammetry
AuNPs-AgCl@PANI-mGCE	AuNPs-AgCl@PANI-modified glassy carbon electrode
AuNR/AuNW/CS	gold nanorod (AuNR) and gold nanowire (AuNW) nanocomposites
Au/4-ATP/AbM/BSA	Au-Gold, 4-ATP—4—aminothiophenol, AbM—monoclonal antibody, BSA—bovine serum albumin
BNQDs	boron nitride quantum dots
BSA	bovine serum albumin
ChOx	cholesterol oxidase
CSPPy-g-C_3_N_4_H^+^	cylindrical spongy shaped polypyrrole
COOH-ZnONPs	carboxylated ZnO nanoparticles (COOH-ZnONPs)
CSA	cTnI specific aptamer
CNT-CH/ITO- CGE	carbon nanotubes-chitosan (CNT-CH)/indium tin oxide (ITO) coated glass electrode
DPV	differential Pulse Voltammetry
DES	deep eutectic solvent
EIS	electrochemical Impedance Spectroscopy
Fc-COFNs	Ferrocene-based covalent organic framework nanosheets
Fc-ECG	a ferrocene derivative
Fe_3_O_4_@SiO_2_	silica coated magnetite nanoparticles
GP	graphite paper
GDF15	growth differentiation factor
GE	graphite electrodes
GCE/poly(oATP)/AuNPs	glassy carbon electrode (GCE) coated with gold nanoparticles (Au-NPs) and poly-o-aminobenzenethiol (poly(oATP)) films
GPTES	3-glycidoxypropyltriethoxysilane
HCNs-GR	graphene supported by hollow carbon balls
HsGDY@NDs	nanodiamonds (NDs) and hydrogen-substituted graphdiyne (HsGDY)
IL-CS	ionic Liquid (1-buthyl-3-methylimidazolium bis (trifluoromethyl sulfonyl)imide)
ITO	indium tin oxide
IX N-CNTs	N-doped carbon nanotubes
LASV	linear sweep anodic stripping voltammetry
LSGE	laser-induced graphene electrodes
LSV	linear Sweep Voltammetry
MWCNTs	multi-walled Carbon Nanotubes
MIPs	molecularly-imprinted polymers
MIP/GWE	molecularly-imprinted polymers onto gold working electrode
MB	methylene blue
MCH	6-mercapto-1-hexanol
MoS2/rGO	modified molybdenum disulfide and reduced graphene oxide
MOF	metal-organic framework
MES	microelectrode system
MGCE	magnetic glassy carbon electrode
MSN	mesoporous silica nanoparticles
MPA	3-mercaptoproponic acid
MIP	molecularly imprinted polymer
MOF-Fc	Ferrocene immobilized metal organic framework
NR	not reported
NFs	nanofibers
N-GNRs-Fe-MOFs@AuNPs	N-doped graphene nanoribbons immobilized fe-based-Metal-organic frameworks deposited with Au nanoparticles
NH2-SWCNT	aminoated single-walled carbon nanotubes
N–ZnO NP	N-doped ZnO nanopolyhedra
NSE	neuron-specific enolase
N-MIP	nano-molecularly imprinted polymer
N-prGO	nitrogen-doped reduced graphene oxide
NH_2_-Rgo/ITO–CGE	aminated Reduced Graphene Oxide (NH_2_-rGO)/ indium tin oxide (ITO)-coated glass electrode
Ox-LDL	oxidized low-density lipoprotein
PAMAM	polyamidoamine
PET	polyethylene terephthalate
PMPC-SH/SAM	thiol-terminated poly (2-methacryloyloxyethyl phosphorylcholine)/self-assembled monolayer
PEI-Fe	Fe (III) phthalocyanine
PPIX	protoporphyrin
PCN	graphitic carbon nitride
pCTAB	cetyltrimethylammonium bromide
PEG	polyethylene glycol
PANI	polyaniline
PMB	polymethylene blue
PTyr	polytyraminesolution
PDMS	polydimethylsiloxane
RTNs	rough-surfaced trimetallic
SPCE	screen-Printed Carbon Electrodes
SPE	graphite screen-printed electrode
SLG	single layer graphene
SWV	square-wave voltammetry
SWASV	square-wave anodic stripping voltammetry
TiO_2_NPs	titanium dioxide nano particles
3-GOPS/anti-adiponectin-ITO-PET	anti-Adiponectin immobilized onto ITO surface with 3-GOPS (3-Glycidoxypropyltrimethoxysilane)
